# Gender differences in outcomes following isolated coronary artery bypass grafting: long-term results

**DOI:** 10.1186/s13019-016-0538-4

**Published:** 2016-09-30

**Authors:** Francesco Nicolini, Antonella Vezzani, Daniela Fortuna, Giovanni Andrea Contini, Davide Pacini, Davide Gabbieri, Claudio Zussa, Rossana De Palma, Tiziano Gherli, RERIC (Registro dell’Emilia Romagna degli Interventi Cardiochirurgici) Investigators

**Affiliations:** 1 Clinical and Experimental Medicine Department, Cardiac Surgery Unit, University of Parma, Parma, Italy; 2 Surgery Department, Cardiac Surgery Unit, Parma Hospital, Parma, Italy; 3 Regional Agency for Health and Social Care, Bologna, Italy; 4 Cardio-Thoracic-Vascular Department, University Hospital S.Orsola-Malpighi, Bologna, Italy; 5 Department of Clinical Cardiology and Thoraco Vascular Surgery, Hesperia Hospital, Bologna, Italy; 6 Department of Cardiology and Cardiac Surgery, Villa Maria Cecilia Hospital, Bologna, Ravenna Italy; 7 Heart Surgery Unit - University of Parma, Via A. Gramsci 14, 43126 Parma, Italy

**Keywords:** Coronary artery disease, Gender, Coronary artery bypass grafting

## Abstract

**Background:**

The main purpose of this study was to evaluate the impact of gender on outcomes after isolated coronary artery bypass grafting, in terms of 5-year rates of overall death, cardiac-related death, myocardial infarction, re-hospitalization, repeat percutaneous or surgical revascularization, stroke, new pacemaker implantation, postoperative renal failure, heart failure and need for long-term care.

**Methods:**

Two propensity-score matched cohorts, each of 1331 patients, undergoing isolated surgical coronary revascularization at the regional public and private centers of Emilia-Romagna region (Italy) from January 1st 2003 to December 31th 2013, were used to compare long-term outcomes of male (5976 patients) versus female gender (1332 patients).

**Results:**

In the matched cohort, males received significantly more bypass grafts (3.0 ± 1.0 vs 2.8 ± 1.0, *p* = 0.001). Left internal mammary artery use and total arterial revascularization were similarly performed in both matched subgroups. Both groups reported similar cumulative rate of all-cause, cardiac-related mortality and stroke at five years. Females experienced significantly higher rate of myocardial infarction, and not significantly higher occurrence of heart failure, and need for long-term care. Males experienced significantly higher rate of cumulative re-hospitalization and higher need for pacemaker implantation. Female gender was not an independent predictor of death at long-term follow-up.

**Conclusions:**

Women are more likely to be readmitted with myocardial infarction and congestive heart failure after CABG but experience survival similar to that observed in men. Female gender was not an independent risk factor for mortality. Prevention of new occurrence of postoperative myocardial infarction and enhancement of complete coronary revascularization should be future endpoints.

## Background

The impact of female gender on clinical outcomes after isolated coronary artery bypass grafting (CABG) has been reported in several studies, with controversial and not definitive results [1–19].

Post-operative outcomes in women undergoing isolated CABG have been intensively studied, due to higher post-operative mortality and morbidity observed in this group of patients [2, 4, 6–12, 14, 15, 17, 18]. However, other studies have shown no difference between male and female patients [1, 3, 5, 13, 16, 19, 20].

Female gender has been clearly reported as an independent predictor of poor post-operative outcomes including mortality after CABG only in a few studies [2, 4, 14, 15]. Moreover, the evidence for the impact of gender in patients who underwent isolated surgical revascularization procedures derives primarily from observational studies based on retrospective registries [3 20], predominantly single center series [1, 4–8, 10–14, 16, 17, 19, 20]. Only few studies investigated a relatively large study population [1, 2, 5, 6, 9, 13, 15, 18] and, with the exception of a few [3–5, 8, 9, 18], most of these studies reported only perioperative or intermediate follow-up outcomes. Finally, another limitation of the previous literature is that it frequently evaluated only the mortality as primary endpoint [6, 8, 10, 11, 13, 15, 17]. There has been less research into long-term differences of other morbidity endpoints and nonfatal events between males and females after isolated CABG [3–5, 9, 18].

Therefore the primary end-point of this multicenter registry study was to evaluate the impact of gender on several outcomes after isolated CABG, comparing 5-year rates of overall death, cardiac related death, myocardial infarction (MI), stroke, postoperative renal failure, overall re-hospitalization, repeat revascularization with CABG or percutaneous coronary intervention (PCI), new pacemaker implantation, new occurrence of heart failure, and need for hospital long-term care in a large study population.

The secondary aim was to evaluate significant risk factors for mortality in this cohort of patients after isolated CABG with multivariate analysis.

## Methods

### Data source

Since 2002, the Agency for Health and Social Care of Emilia-Romagna (ER) region has maintained the RERIC Registry (Registro dell’Emilia Romagna degli Interventi Cardiochirurgici), a prospective regional database collecting pre-, intra- and postoperative data from all the patients operated on in the six hospitals able to perform cardiac surgery (two public University Hospitals and four private Hospitals). The rationale and methodology of RERIC have been published previously [21]. This registry is based on current clinical practice, but the requirement for individual patient consent was waived because of the retrospective design of the study and because data were collected from routine care. All data were anonymized and de-identified prior to analysis by the central statistical laboratory of the Regional Agency for Health and Social Care. The protocol of the study is in accordance with the Declaration of Helsinki.

### Study population

From January 1st, 2003 to December 31th, 2013, data of all the patients undergoing CABG were collected in the RERIC Registry. Exclusion criteria were: emergency, cardiogenic shock, associated valve surgery procedures, major aortic surgery and supra-aortic vessels disease requiring surgery. After these exclusions, we filtered 9382 patients subjected to isolated CABG. Additional exclusion criteria were patients not resident in ER (administrative follow-up not feasible) and the presence of incomplete information about baseline and procedural characteristics. The remaining 7308 patients, 5976 males and 1332 females, were followed through December 2014.

### Procedures

Decisions about the type of treatment were taken according to local practices and there were no standard regional protocols. The choice of CABG technique has been previously described [22]. Follow-up angiography was not performed routinely in either group of patients.

### Definition of the outcomes

All-cause death included overall mortality occurring during the index hospital admission or thereafter. Cardiac death was defined as any death due to a cardiac cause (e.g., MI, low output failure, fatal arrhythmia), procedure-related deaths and death of unknown cause. Stroke included complications at the index admission and further hospital admissions with stroke as principal diagnosis. Postoperative renal failure was defined as any hospital admission occurring after the index procedure with the principal diagnosis of renal failure, excluding from the analysis all the patients with a preoperative creatinine levels ≥2.0 md/dL. Overall re-hospitalization was defined as any hospital admission after the index procedure due to a cardiac cause (e.g. MI, repeat PCI, repeat CABG, new pacemaker implantation, new occurrence of heart failure, or need for hospital long-term care). Acute MI was defined as any hospital admission occurring after the index procedure with a principal diagnosis of MI. Repeat PCI was defined as any percutaneous coronary intervention during follow-up, treating a luminal stenosis occurring in the same coronary vessel treated at the index procedure, or treating other native vessel stenosis not previously revascularized. Repeat CABG was defined as any new surgical revascularization occurring during the follow-up. New pacemaker implantation was defined as any device implantation performed in the follow-up. New occurrence of heart failure was defined as any hospital admission occurring after the index procedure with the principal diagnosis of heart failure. Long-term care is defined as the set of services required by a patient whose functional, physical and cognitive capacities are reduced for an extended period of time, and who is therefore not autonomous in carrying out daily activities.

### Statistical analysis

Prevalence of risk factors and demographic and clinical features of the patients in both groups were compared by the Chi-square test and Fisher’s exact test.

Propensity score (PS) matching was used to reduce the effect of gender-selection bias. PS, was estimated by multivariate logistic regression analysis with a binary dependent variable representing female versus male. Independent variables included: age, obesity, unstable angina, left ventricular ejection fraction, previous myocardial infarction, pre-operative serum creatinine, diabetes, systolic PA pressure, chronic pulmonary disease, New York health Association functional classification (NYHA), extracardiac arteriopathy, neurological dysfunction disease, previous cardiac surgery, number of diseased vessels and Euroscore. Patients were matched on the logit of the PS using a caliper of width equal to 0.25 standard deviation of the logit of PS.

To detect imbalances in baseline covariates, standardized differences were used. Standardized differences represent the difference in means between the two groups in units of standard deviation. They do not depend on the unit of measurement and are not influenced by sample size. Standardized differences of less than 0.10 (10 %) are likely to indicate a negligible imbalance between the two groups.

Kaplan-Meier estimates were used to plot the rates of the long-term adverse events, and differences between risk curves were assessed using the Klein-Moeschberger test for matched pairs.

To evaluate the effect-size in case of moderate difference of outcome rates between the cohorts, statistical power was estimated and the necessary sample size at 90 % CI was calculated.

Independent predictors of 5-year mortality risk were estimated using a stepwise multivariable Cox proportional hazards model, with robust standard errors to account for clustering in matched pairs including treatment, and individual covariates such as all pre- and intraoperative variables available.

All the analyses were performed with SAS version 9.3

## Results

**Fig. 1:**
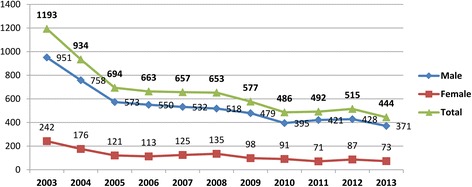
Isolated CABG trend in both genders over years in Emilia-Romagna region (Italy)

A constant reduction in the number of overall isolated elective CABG was observed in our registry between 2003 and 2013, although this reduction occurred mainly in the male patients subgroup (Fig. 1).

The entire study cohort showed patient risk profiles significantly different in the two groups (Table 1). Females represented 18.2 % of the overall isolated CABG procedures in the study period. They were older, and had a higher prevalence of diabetes and unstable angina. On the other hand, the male group had more patients with chronic obstructive pulmonary disease, previous CABG, depressed left ventricular ejection fraction and presented with a more complex and severe coronary artery disease (Table 1).

**Table 1:** Baseline characteristics of the patients according to gender

Patients’ Characteristics	Male (*N* = 5976)	%	Female (*N* = 1332)	%	*p*-value	Standardized differences
<=70 year	3684	61.6	555	41.7	<.0001	−0.408
70-80 year	2255	37.7	699	52.5	<.0001	0.3
>80 year	264	4.4	124	9.3	<.0001	0.194
BMI > =30: obesity	1244	20.8	297	22.3	0.2309	0.036
Unstable angina	459	7.7	123	9.2	0.0583	0.056
Ejec. fraction < =30 %	166	2.8	25	1.9	0.0624	−0.06
Ejec. fraction 30 %-50 %	1550	25.9	304	22.8	0.0182	−0.073
Previous myocardial infarction	1804	30.2	417	31.3	0.4221	0.024
Serum Creatinine > =2 mg/dl	158	2.6	30	2.3	0.4142	−0.025
Diabetes	1788	29.9	481	36.1	<.0001	0.132
Systolic PA pressure > 60 mmHg	7	0.1	1	0.1	0.6746	−0.014
Chronic pulmonary disease	352	5.9	54	4.1	0.0082	−0.085
NYHA III-IV	628	10.5	154	11.6	0.261	0.034
Extracardiac arteriopathy	1396	23.4	306	23	0.7624	−0.009
Neurological dysfunction disease	109	1.8	31	2.3	0.2255	0.035
Previous cardiac surgery	137	2.3	16	1.2	0.0119	−0.083
Single-vessel disease	572	9.6	169	12.7	0.0007	0.099
Double-vessel disease	2328	39	527	39.6	0.6805	0.012
Triple-vessel disease	3076	51.5	636	47.7	0.0139	−0.075
Previous PCI	934	15.6	212	15.9	0.7947	0.008
Previous CABG	112	1.9	9	0.7	0.0019	−0.107
EuroScore: 0-4	3385	56.6	441	33.1	<.0001	−0.487
EuroScore: 5-6	1214	20.3	376	28.2	<.0001	0.185
EuroScore: 7-9	739	12.4	339	25.5	<.0001	0.339
EuroScore: > = 10	171	2.9	90	6.8	<.0001	0.183
OPCABG	483	8.1	114	8.6	0.5801	0.017

*BMI* body mass index, *LVEF* left ventricular ejection fraction, *NYHA* New York Health Association, *PCI* percutaneous coronary intervention, *CABG* coronary artery bypass grafting, *OPCABG* Off-pump coronary artery bypass grafting

The entire male group received 3.1 ± 1.1 bypass grafts, compared with 2.8 ± 1.0 in the female group (*p* < 0.0001). Left internal mammary artery (LIMA) was used in 92.3 % of the entire male group versus 88.6 % of females (*p* = 0.0002). Coronary revascularization completely performed with arterial grafts was reported in 23 % of males and 22.6 % of females (*p* = 0.602). The median duration of follow-up in the entire study cohort was 95.4 ± 39.6 months in the male subgroup and 97.6 ± 39.5 months in the female subgroup.

Matching on estimated PS made available a matched cohort of 2662 patients, with similar demographic, clinical and angiographic risk profiles, 1331 in each group (Table 2).

**Table 2:** Propensity score-adjusted characteristics of the patients according to gender

Patients' Characteristics	Male (*N* = 1331)	%	Female (*N* = 1331)	%	*p*-value	Standardized differences
<=70 year	557	41.8	555	41.7	0.8897	−0.003
70–80 year	693	52.1	698	52.4	0.7679	0.008
>80 year	125	9.4	124	9.3	0.9372	−0.003
BMI > =30: obesity	282	21.2	297	22.3	0.3173	0.027
Unstable angina	117	8.8	123	9.2	0.6473	0.016
Ejec. fraction < =30 %	20	1.5	25	1.9	0.398	0.029
Ejec. fraction 30 %-50 %	290	21.8	304	22.8	0.3474	0.025
Previous myocardial infarction	413	31	417	31.3	0.8174	0.006
Serum Creatinine > =2 mg/dl	26	2	30	2.3	0.5637	0.021
Diabetes	437	32.8	480	36.1	0.0795	0.068
Systolic PA pressure > 60 mmHg	2	0.2	1	0.1	0.5637	−0.022
Chronic pulmonary disease	59	4.4	54	4.1	0.5737	−0.019
NYHA III-IV	155	11.6	154	11.6	0.9438	−0.002
Extracardiac arteriopathy	294	22.1	306	23	0.4442	0.022
Neurological dysfunction disease	25	1.9	31	2.3	0.4054	0.031
Previous cardiac surgery	13	1	16	1.2	0.5637	0.022
Single-vessel disease	162	12.2	169	12.7	0.5689	0.016
Double-vessel disease	517	38.8	527	39.6	0.5611	0.015
Triple-vessel disease	652	49	635	47.7	0.3139	−0.026
Previous PCI	208	15.6	212	15.9	0.7835	0.008
Previous CABG	7	0.5	9	0.7	0.593	0.019
EuroScore: 0-4	442	33.2	441	33.1	0.9028	−0.002
EuroScore: 5-6	383	28.8	376	28.2	0.6451	−0.012
EuroScore: 7-9	343	25.8	339	25.5	0.792	−0.007
EuroScore: > = 10	81	6.1	89	6.7	0.4576	0.025
OPCABG	128	9.6	114	8.6	0.3452	−0.037

*BMI* body mass index, *LVEF* left ventricular ejection fraction, *NYHA* New York Health Association, *PCI* percutaneous coronary intervention, *CABG* coronary artery bypass grafting, *OPCABG* Off-pump coronary artery bypass grafting

The matched male group received significantly more bypass grafts than the matched female group (3.0 ± 1.0 vs 2.8 ± 1.0, *p* = 0.001). Left internal mammary artery was used in 90.5 % of the matched male group versus 88.6 % of females (*p* = 0.114). Total arterial revascularization was similarly performed in both matched cohorts (23.2 % of males versus 22.6 % of females (*p* = 0.702)). Table 3 summarizes the results of the main endpoints at 30-days follow-up. Both groups reported similar outcomes except for new renal failure rate and re-hospitalization rate which were significantly higher in male patients. However, as reported in Table 3, with the exception of the renal failure, the calculation of the statistical power demonstrated that the differences between cohorts were very small and the probability of highlighting a real difference was less than 50 %.

**Table 3:** Thirty day outcomes in propensity matched population

	At 30 days						
	Male		Female		Log-rank Test: *p*-value	Statistical power	Sample size calculation at 90 % CI and 80 % statistical power
	*N*°	%	*N*°	%			
All-cause death	17	1.30 %	24	1.80 %	0.27	42.7 %	7726
Cardiac death	15	1.10 %	21	1.60 %	0.314	47.5 %	6588
MI	2	0.20 %	2	0.20 %	0.997	10.0 %	na
Stroke	1	0.10 %	2	0.20 %	0.561	24.6 %	18520
Renal failure	4	0.30 %	0	0.00 %	0.046	88.2 %	2058

In the matched cohort, mean follow-up was 94 ± 38.5 months in the male group and 97.6 ± 39.5 months in the female group. Table 4 summarizes the results of the main endpoints at 5-year follow-up. For all outcomes, with the exception of stroke, renal failure and redo CABG, the numerosity of the two matched cohorts ensured a good power of the study, ranging from a minimum of 80 %, for all cause death, to a maximum of 99 % in the case of cardiac heart failure.

**Table 4:** Five-years outcomes in propensity matched population

Outcomes at 5 years	Male (*N* = 1331)	Female (*N* = 1331)	Moeschberger Test *p*-value	Statistical power	Sample size calculation at 90 % CI and 80 % statistical power
All-cause death	15.8	14.7	0.1745	80 %	2592
Cardiac death	6.2	7.8	0.2623	85.90 %	1784
Myocardial infarction	4.8	7.3	0.0446	>0.99	592
Stroke	5.4	5.3	0.8445	10 %	>100000
Renal failure	4.6	4.6	0.34	10 %	>100000
Re-hospitalization	47.4	45.9	0.0499	97.20 %	1096
Long-term care	9.3	11.1	0.0849	86.30 %	2192
Cardiac heart failure	8.3	10.9	0.0633	98.30 %	1254
Repeat PCI	6.5	8	0.2498	86.80 %	2156
Repeat CABG	0.1	0.2	0.1573	39.50 %	8716
Pacemaker implantation	3.2	1.5	0.0047	>0.99	486

**Fig. 2:**
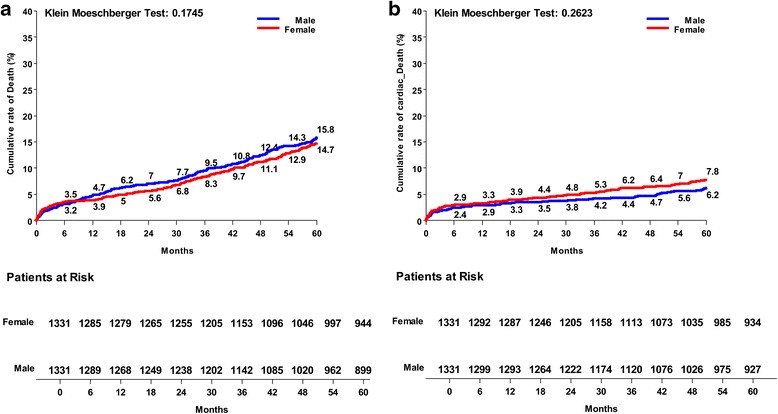
Kaplan Meier risk curves between males and females operated on isolated CABG: **a** cumulative rate of all-cause death; **b** cardiac-related mortality

**Fig. 3:**
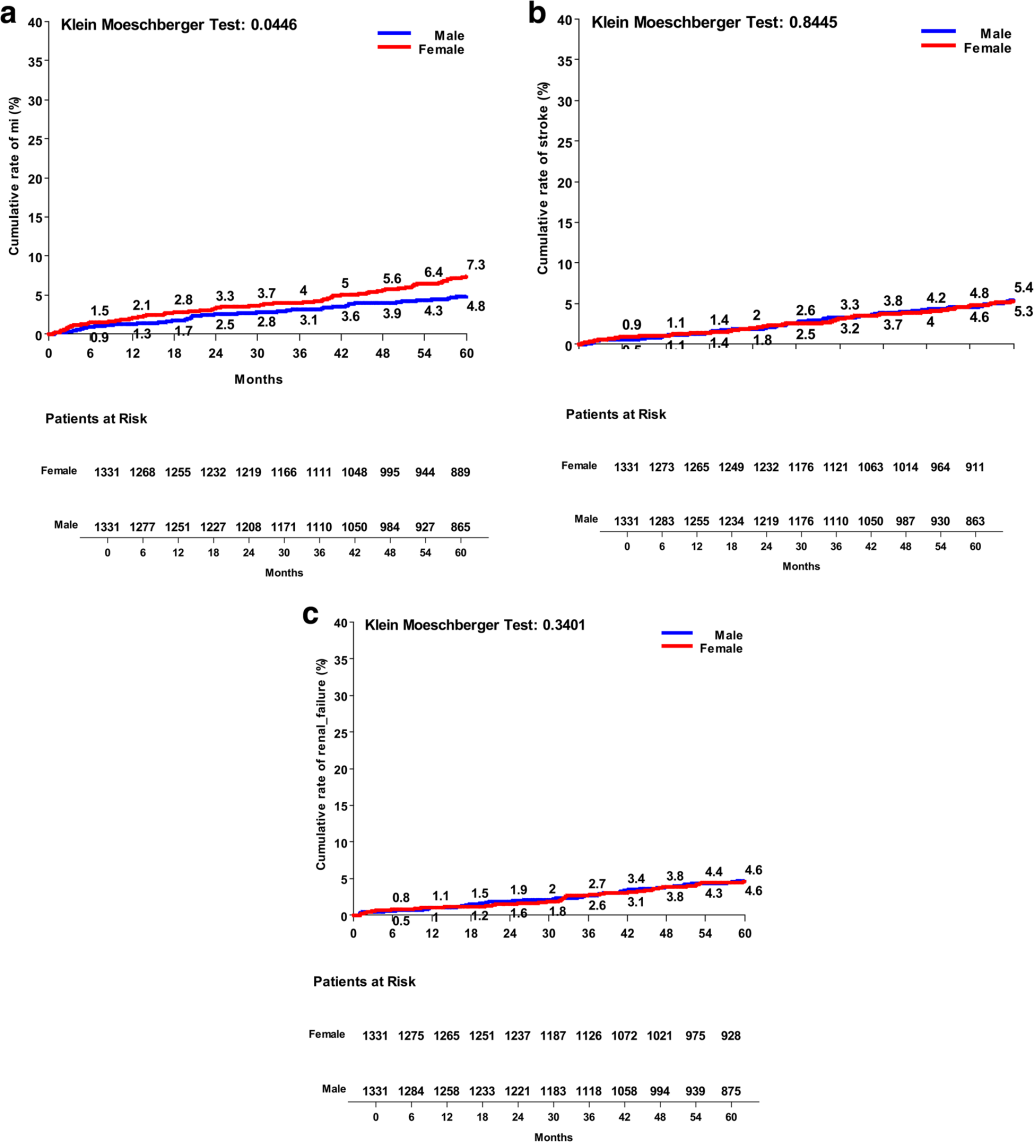
Kaplan Meier risk curves between males and females operated on isolated CABG: **a** myocardial infarction rate; **b** stroke rate; **c** new occurrence of postoperative renal failure

**Fig. 4:**
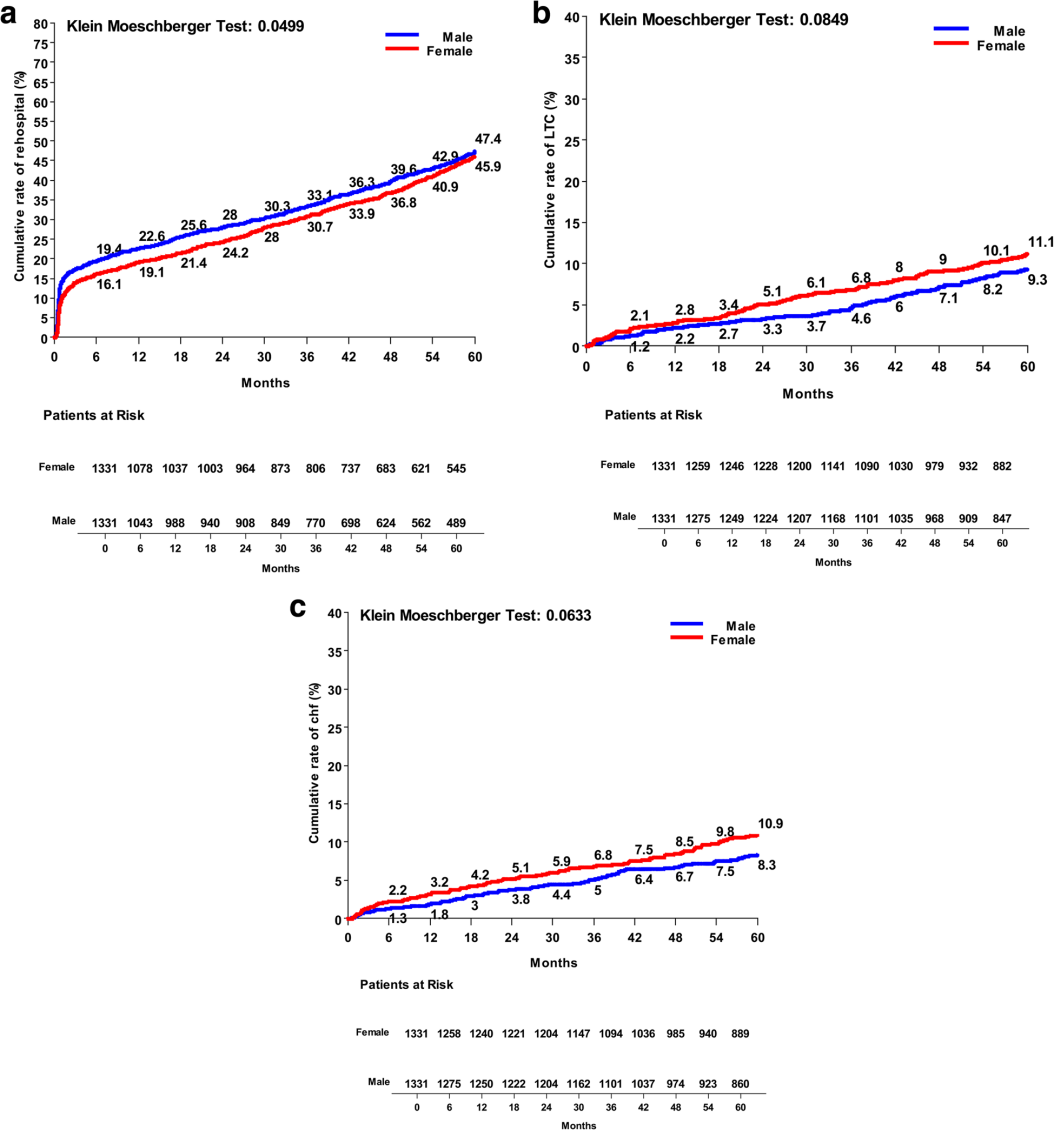
Kaplan Meier risk curves between males and females operated on isolated CABG: **a** overall re-hospitalization rate; **b** need for long-term care; **c** new occurrence of heart failure

**Fig. 5:**
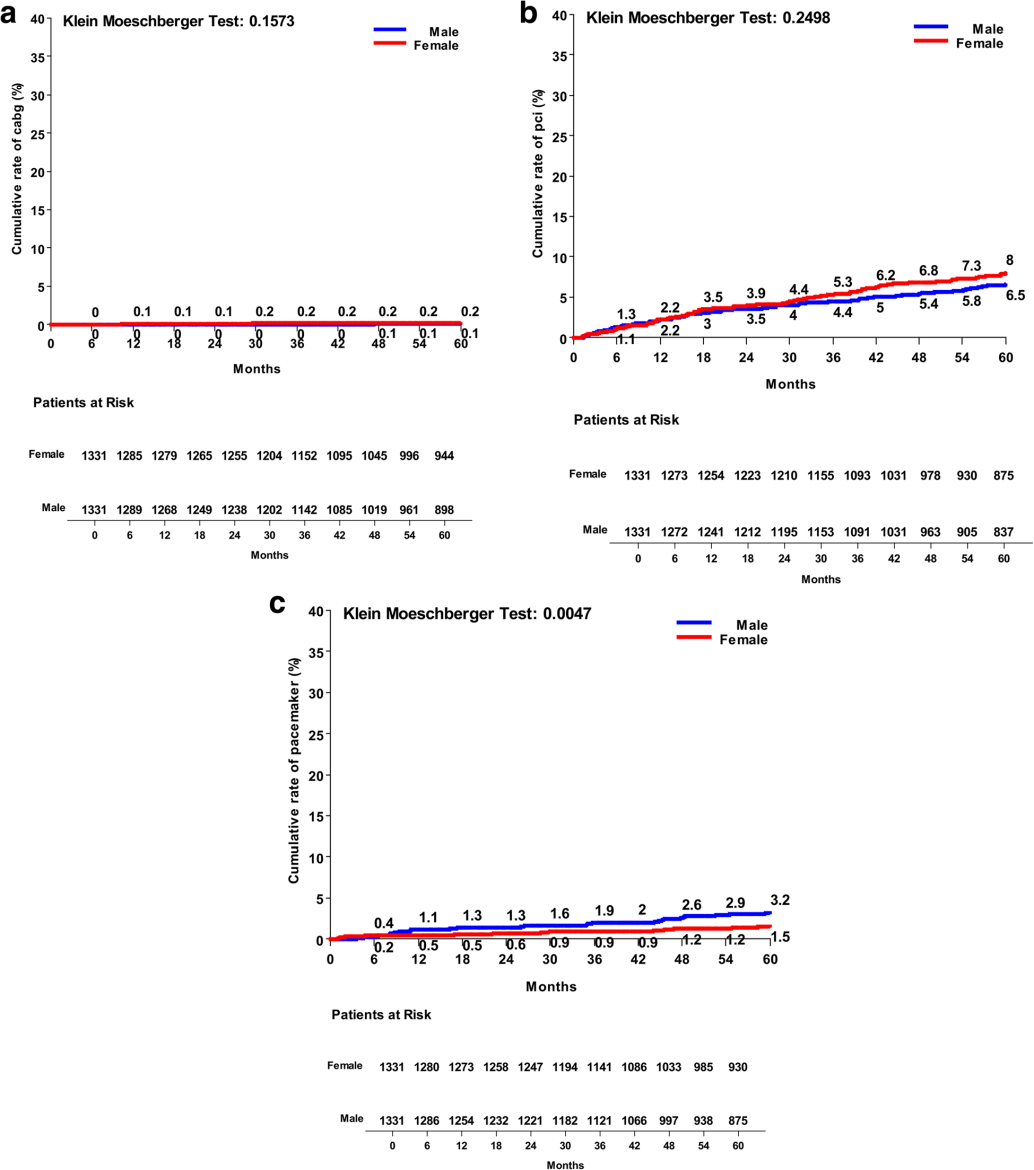
Kaplan Meier risk curves between males and females operated on isolated CABG: **a** repeat PCI; **b** RE-DO CABG; **c** new pacemaker implantation

Kaplan Meier risk curves for the matched population at five years were reported. Both groups reported similar cumulative rates of all-cause death (Fig. 2a) and cardiac-related mortality at five years (Fig. 2b). Females experienced a significantly higher rate of MI (Fig. 3a), and similar occurrence of stroke (Fig. 3b) in the follow-up. No differences were found for new occurrence of postoperative renal failure between groups (Fig. 3c), whereas males experienced a significantly higher cumulative rate of re-hospitalization for cardiac causes (Fig. 4a). The female group confirmed greater occurrence of heart failure episodes at five years (Fig. 4b), and greater need for hospital long-term care, although without any statistical significance (Fig. 4c). No difference was found either for repeat PCI (Fig. 5a), or for RE-DO CABG (Fig. 5b), whereas males reported a higher rate of definitive pacemaker implantation (Fig. 5c).

Multivariate analysis showed the following significant independent predictors of mortality at 5 years: age 70–80 year (HR 1.8, LCI 1.4, UCI 2.1, *p* < 0.0001), age >80 year (HR 2.5, LCI 1.9, UCI 3.4, *p* < 0.0001), chronic pulmonary disease (HR 1.4, LCI 1.0, UCI 1.9, *p* = 0.03), serum creatinine level > =2 mg/dl (HR 2.2, LCI 1.4, UCI 3.3, *p* = 0.0002), LVEF 30-50 % (HR 1.4, LCI 1.1, UCI 1.6, *p* = 0.0005), and diabetes (HR 1.6, LCI 1.3, UCI 1.8, *p* < 0.0001). Female gender was not an independent predictor of death at long-term follow-up.

## Discussion

The impact of gender on clinical outcomes after CABG remains controversial, and because there are many discrepancies between studies [1–20], requires further review.

The main findings of this study are that, after propensity matching, males group received significantly more bypass grafts than the matched females group. LIMA was used in a similar percentage of patients in both genders and total arterial revascularization was performed in similar numbers in both matched cohorts. At five years, both groups reported similar cumulative rate of all-cause death and cardiac-related mortality. Females experienced a significantly higher rate of MI, and similar occurrence of stroke in the follow-up. No differences were found for new occurrence of postoperative renal failure between groups. In particular, no difference was found neither for repeat PCI, nor for RE-DO CABG, whereas males reported a higher need for new pacemaker implantation. Males experienced a significantly higher cumulative rate of re-hospitalization, whereas the female group showed greater occurrence of heart failure episodes at five years and greater need for hospital long-term care, although without any statistical significance. Finally, multivariate analysis of significant predictors of mortality in the overall population failed to demonstrate that female gender was an independent predictor of death at long-term follow-up.

It is widely recognized that women presented more frequently at higher age and with a worse and more complex clinical condition than men [23, 24]. Moreover the smaller body size of women is thought to be related to the smaller diameter epicardial coronary arteries. This, along with a greater preponderance of metabolic syndrome [24], and potential differences in neuro-humoral responses in the vasculature may also contribute to the reported adverse outcomes in the post-operative period.

Several recent reports have confirmed increased post-operative mortality and morbidity in women after isolated CABG [24–27]. A meta-analysis of 20 studies comparing 966,492 patients also reported that women had increased risk for short-term, midterm and long-term mortality compared with men. Mortality remained independently associated with female gender despite propensity score-matched analysis of outcomes [25]. The international randomized controlled IMAGINE study [27] included 2553 consecutive patients (2229 men and 324 women) with a LVEF >40 % who underwent isolated CABG, with the aim to determine sex differences in long-term outcomes. The composite endpoint comprised death, MI, cerebrovascular event, angina, revascularization and congestive heart failure. After adjusting for potential confounders, female sex became a non-significant predictor for prognosis, possibly due to the small sample size of women. The authors concluded that “definite answers regarding sex-differences in long-term outcome after CABG should come from future pooling of studies comprising a larger number of women”.

Our study is one of few large-scale clinical multicenter retrospective studies comparing the long-term mortality, morbidity outcomes and nonfatal events between females and males undergoing isolated CABG. Another strenght point of this study is that, for all 5-year outcomes, with the only exception of stroke, renal failure and redo CABG, the numerosity of the two matched cohorts ensured a good power of the study, ranging from a minimum of 80 %, for all cause death, to a maximum of 99 % in the case of cardiac heart failure.

Like previous studies our entire study cohort also showed significantly different patient risk profiles in the two groups, but multiple factors could explain the differences in our post-operative outcomes compared to other prior reports [24–27]. Close PS matching for example resulted in removal of referral bias and correction of baseline differences. Moreover, this registry presents data from high volume tertiary care referral centers, where surgeons experience and modern post-operative critical care may also partially explain similar outcomes for mortality and several morbidity endpoints between the two genders. In our PS matched population, although the male group received significantly more bypass grafts than the matched female group, widely-used modern techniques such as LIMA grafting and total arterial revascularization were performed in similar numbers in both genders. This strategy may explain the similar overall and cardiac-related mortality rates reported in both groups at five years. It is also likely that the significantly lower total number of grafts performed in females, with a consequent less complete revascularization, explains other worse morbidity outcomes such as higher rates of MI and readmission for heart failure in the female group. Unfortunately, data on graft targets which could be used to verify this inference are not available. Our study also shows that these events occurred significantly in females even though they are less likely to have poor ventricular function preoperatively. This is consistent with the results of other studies reporting that women had similar or improved long-term survival compared with men despite the gender difference in readmission rates for heart failure [5, 28]. Moreover in our study we found that females require hospitalization for long-term care more frequently, consistent with the results of Asch et al. [29]. These authors reported that women are more likely to receive treatment for chronic diseases, but less likely to receive recommended treatments for acute diseases. Our data also confirm the results of Kurlansky et al. [30] who found that, even where optimal grafting practice takes place, women still received fewer grafts than men and experienced a statistically significant higher rate of late myocardial infarction, and that total arterial coronary revascularization in women improved the gender disparity only in terms of mortality outcomes.

Previous studies showed different results in terms of risk of adverse outcomes in women who underwent coronary revascularization with off-pump coronary artery bypass grafting (OPCABG), compared to men. Cartier et al. [8] showed that above 65 years of age men and women had a comparable overall survival (*p* = 0.7) whereas fewer than 65 women had a lower survival than men (*p* = 0.001). On the other hand, Puskas et al. [9] compared in-hospital major adverse cardiac events (MACE) and long-term survival after OPCABG vs on-pump CABG. Women disproportionately benefited from OPCABG in operative mortality (*p* = 0.04). Odds of death for women on CPB were higher than for women treated with OPCABG (OR, 2.07, *p* = 0.005). However, during the 10-year follow-up, OPCABG and on-pump CABG result in similar survival, regardless of gender. Uva et al. [10] reported that female gender was not an independent risk factor for mortality or major morbidity in an unselected patient population undergoing OPCABG. In our experience there were no significant differences in the percentage of off-pump operations between males and females in the initial unmatched study population. Moreover off-pump technique did not demonstrate significant statistical differences in terms of mortality between matched cohorts. Probably the small size of this matched subgroups did not allow for significant conclusions. Finally off-pump technique had already been included as a covariate in the multivariate analysis, but it did not result an independent risk or protective factor (HR 1.1 *p* = 0.364).

Although there are conflicting results about gender differences in stroke rate after CABG [5, 24], post-operative stroke has not traditionally been related to gender, and this is also confirmed in our matched analysis. However, at 5 years the evaluation of the effect-size of stroke rate between both cohorts showed an evident lack of statistical power and a consequent uncorrect evaluation of this outcome.

In our study males experienced significantly higher permanent pacemaker implantation in the follow-up. A possible explanation is that men presented with a more complex and diffuse coronary artery disease, and in fact it has been reported that specific severe distribution of coronary lesions is closely correlated with conduction defects [31]. Moreover, Yesil et al. reported that coronary revascularization, even when completely performed, as in the majority of our male subgroup, has little, if any, impact on reversibility of conduction defects [31].

The limitations of a retrospective registry study should be noted. The entire study cohort showed significantly different patient risk profiles in the two groups. Although we tried to rigorously adjust selection bias using propensity score-based analysis, unmeasured confounders and hidden biases may have affected our results. Moreover at 5 years the lack of statistical power of some variables as stroke, renal failure and redo CABG did not ensure a correct evaluation of these outcomes. Finally limitations of the present study include lack of echocardiographic follow-up, graft-patency, cause-of-death data and quality-of-life measures. These parameters could have differed between women and men.

## Conclusions

The present study highlights the finding that, despite equivalent overall and cardiac related mortality for both genders after CABG surgery, MI incidence and occurrence of heart failure for women are higher than for men. There are operative disparities in the number of bypass grafts that women receive, which results in a lower percentage of complete revascularization and which could partially explain these differences in morbidity. The similar rates of use of LIMA and total arterial coronary revascularization in both genders is probably a protective factor only in terms of mortality outcomes. Female gender was not found to be an independent adverse risk factor for mortality associated with CABG.

Given the associated conditions in women, we need to ensure future efforts to maximize the completeness of revascularization and the prevention of follow-up morbidity in order to optimize women’s postoperative outcomes to rates observed for men. On the basis of our data, decisions regarding surgical revascularization should not be based on gender.

## Abbreviations

CABG: Coronary artery bypass grafting
ER: Emilia-Romagna
LIMA: Left internal mammary artery
MACE: Major adverse cardiac events
MI: Myocardial infarction
NYHA: New York Health Association
OPCAB: Off pump coronary artery bypass grafting
PCI: Percutaneous coronary intervention
PS: Propensity score
RERIC: Registro dell’Emilia Romagna degli Interventi Cardiochirurgici
